# Gestion de la rechute d’une tuberculose naso-sinusienne primaire

**DOI:** 10.11604/pamj.2020.36.84.13067

**Published:** 2020-06-12

**Authors:** Adil Zegmout, Ahmed Rouihi, Abdelhalim Boucaid, Younes Amchich, Hicham Souhi, Hanane El Ouazzani, Ismail Abderrahmane Rhorfi, Ahmed Abid

**Affiliations:** 1 Service de Pneumologie, Hôpital Militaire d'Instruction Mohamed V, Rabat, Maroc,; 2 Service d’ORL, Hôpital Militaire d'Instruction Mohamed V, Rabat, Maroc

**Keywords:** Tuberculose, tuberculose extra-pulmonaire, tuberculose naso-sinusienne, rechute, Tuberculosis, extra-pulmonary tuberculosis, naso-sinusal tuberculosis, recurrence

## Abstract

La tuberculose naso-sinusienne primaire est une affection relativement rare voir exceptionnelle. La présentation clinique est polymorphe et non spécifique. Le diagnostic définitif repose sur l’examen anatomopathologique et l’examen mycobactériologique d’une pièce biopsique de la lésion. Son pronostic est favorable sous une antibiothérapie antituberculeuse classique et précoce. Cependant, notre observation rapporte une rechute de cette localisation rare chez une patiente immunocompétente malgré un traitement antituberculeux bien adaptée initialement et avec une bonne observance. La rechute a été attribuée à un sous dosage en rifampicine. Les auteurs insistent sur la démarche diagnostique, étiologique et sur la gestion thérapeutique de cette rechute. Notre expérience pourrait aider les cliniciens à mieux gérer cette situation inhabituelle.

## Abstract (en)

Primary naso-sinusal tuberculosis (TB) is a relatively rare or exceptional disorder characterized by polymorphic or non-specific clinical manifestation. Diagnosis is based on anatomo-pathological examination and mycobacteriology test of biopsy specimen. Predictor of good outcome is early conventional anti-tuberculous antibiotic therapy. However, our study reports recurrence at this rare site in an immunocompetent patient despite early suitable TB treatment and good adherence with therapy. Relapse was correlated with underdosing of rifampicin. This study highlights the diagnostic, etiological and therapeutic management of this relapse. Our experience could help clinicians to better manage this uncommon condition.

## Introduction

La tuberculose naso-sinusienne est une infection des cavités nasales et des sinus de la face par le *Mycobacterium tuberculosis*. Quelques rares cas ont été rapportés dans la littérature [1,2]. Elle est caractérisée par une présentation clinique polymorphe et non spécifique, posant souvent un problème de diagnostic différentiel [2,3]. La revue de littérature n’a trouvé aucun cas de rechute de cette localisation rare, l’évolution est généralement favorable sous traitement antibacillaire. Les auteurs rapportent la gestion d’une rechute d’une tuberculose naso-sinussienne primaire chez une patiente immunocompétente. A travers cette observation, nous allons insister sur le défi diagnostic posé par la rareté de cette pathologie et ses symptômes non spécifiques, nous allons évoquer la démarche étiologique pour rechercher les facteurs expliquant cette rechute, et nous allons partager notre expérience concernant la gestion thérapeutique adaptée face à cette situation inhabituelle.

## Patient et observation

Il s’agit d’une patiente âgée de 64 ans, asthmatique traitée par corticoides inhalés et bronchodilatateurs, ayant un antécédent de tuberculose naso-sinusienne primaire évoquée devant une rhinorrhée purulente et une obstruction nasale avec à la tomodensitométrie (TDM) un comblement des sinus de la face. L’étude histologique des biopsies des lésions de la muqueuse nasales constatées à l’examen oto-rhino-laryngologie (ORL) conclut à des remaniements inflammatoires granulomateux spécifiques évoquant en premier lieu une origine tuberculeuse. L’évolution était favorable sous traitement antibacillaire bien conduit pendant 6 mois et associant l’isoniazide, la rifampicine, la pyrazinamide et l’ethambutol. La patiente n’avait pas d’antécédent de diabète ni de malabsorption. Deux ans après, la patiente présentait une rhinorrhée mucopurulente bilatérale associée à une obstruction nasale avec hyposmie, évoluant dans un contexte d’apyrexie et de conservation de l’état général. La patiente avait reçu plusieurs traitements antibiotiques (amoxicilline - acide clavulanique, macrolides) sans amélioration de la symptomatologie. A son admission, l’examen clinique trouvait une patiente en bon état général et apyrétique. Les aires ganglionnaires étaient libres.

**Figure 1:**
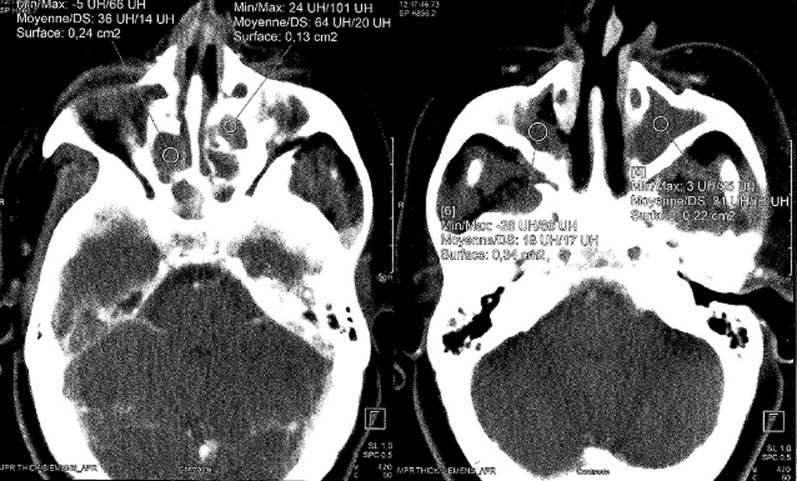
TDM naso-sinusienne: coupes axiales en fenêtres parenchymateuses après injection du produit de contraste mettant en évidence un comblement subtotal hypodense et hétérogène des sinus maxillaires et des cellules ethmoïdales (cercles)

**Figure 2:**
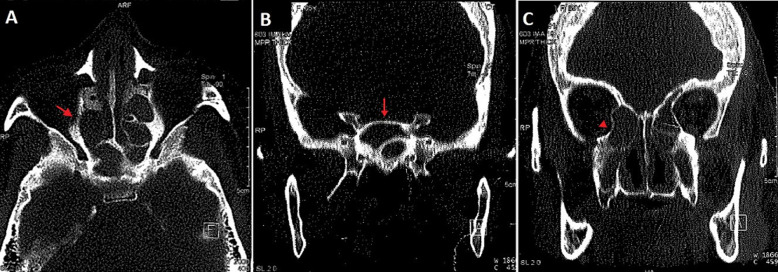
TDM naso-sinusienne: coupe axiale (A) avec reconstructions coronales (B et C) en fenêtres osseuses mettant en évidence un aspect soufflé (flèche) avec amincissement et lyse par endroits (triangle) des parois des sinus de la face et des cellules ethmoïdales

**Figure 3:**
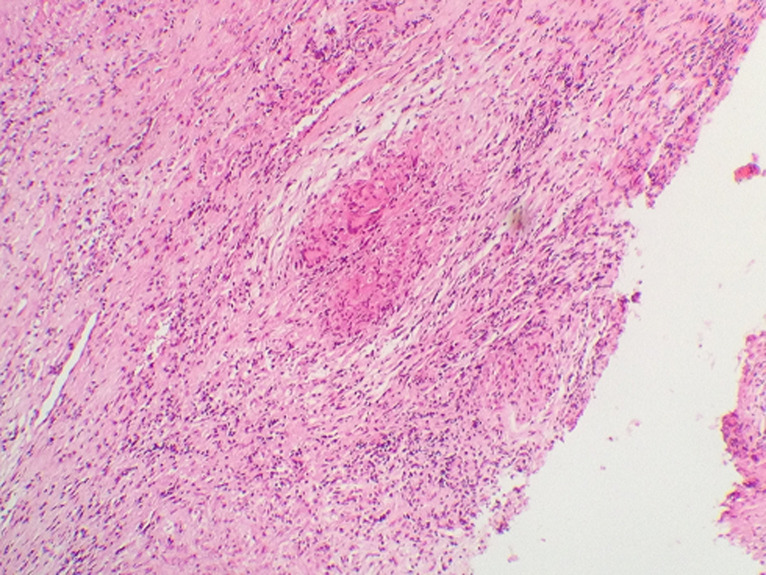
coupe anatomo-pathologique montrant un processus inflammatoire granulomateux

**Figure 4:**
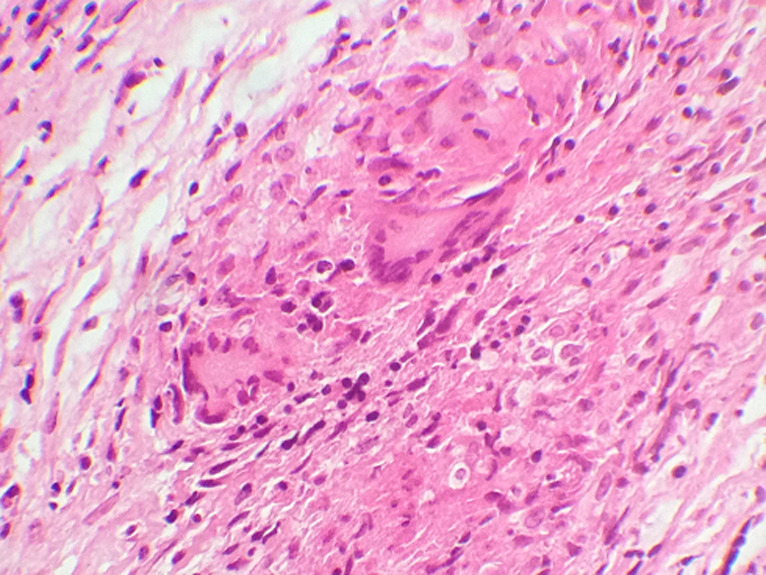
processus fait de follicules épithélio-gigantocellulaires

Le bilan biologique notait un syndrome inflammatoire avec une vitesse de sédimentation à 88mm à la 1^ère^ heure, l'hémogramme était normal avec des globules blancs à 6800 éléments/mm^3^, hémoglobine à 12g/dl, les plaquettes à 350 000 éléments/mm^3^. L’intradermoréaction à la tuberculine était positive à 15mm. La sérologie VIH était négative. La radiographie du thorax était normale. Le scanner naso-sinusienne montrait un comblement de tous les sinus de la face par un matériel de densité tissulaire (Figure 1), avec une lyse par endroit et soufflure des parois des cellules ethmoïdales (Figure 2), en plus d’une légère déviation de la paroi interne de l’orbite droit refoulant le muscle droit interne. L’endoscopie nasale montrait des sécrétions épaisses provenant du méat moyen avec une muqueuse congestive. Des biopsies ont été réalisées au niveau de la muqueuse nasale et de la région du méat moyen. L’étude histologique du tissu nasal a objectivé la présence d’un processus inflammatoire non spécifique. Une méatotomie avec extraction du matériel intra-sinusien était effectuée. L’étude anatomo-pathologique montre un processus inflammatoire granulomateux (Figure 3), ce processus est fait de follicules épithélio-gigantocellulaires (Figure 4).

Le prélèvement bactériologique du pus à la recherche des bacilles acido-alcolo-résistants est revenue positive à l’examen direct avec isolement d’un *Mycobacterium tuberculosis* à la culture sur milieu spécifique. Un test de sensibilité était réalisé et n’a pas montré de résistance à l’isoniazide, ethambutol et à la rifampicine. La patiente était mise sous traitement antibacillaire associant isoniazide (300mg/j), rifampicine (600mg/j), ethambutol (1200mg/j) et pyrazinamide (1600mg/j). Un dosage sérique des antibacillaires était réalisé après 10 jours de la prise du traitement. Le protocole comportait un prélèvement sanguin à la 2^è^ et 6^è^ heure pour la rifampicine, à la 3^è^ heure pour l’isoniazide, et à la 2^è^ heure pour la pyrazinamide. Ce dosage avait objectivé un sous dosage en rifampicine à 4,82mg/l (valeur normale entre 8 et 24mg/l), les résultats de dosage sérique des autres médicaments étaient inclus dans la fourchette thérapeutique. Une supplémentation de 150mg par jour en rifampicine était décidée. Le dosage sérique de contrôle trouvait une valeur de 11,5mg/l. Le traitement était bien toléré cliniquement et biologiquement avec un bilan hépatique qui est resté normale tout au long du traitement. L’évolution clinique et endoscopique était favorable permettant l’arrêt du traitement à 9 mois. Un suivi de 5 ans n’a pas montré de récidive des symptômes.

## Discussion

La tuberculose est un problème de santé publique majeur dans le monde malgré les nombreuses stratégies de lutte antituberculeuse (LAT). À l’échelle mondiale, on estime que 10 millions de personnes ont contracté la tuberculose en 2019 dont 8,6% sont vivant avec VIH [4]. La tuberculose (TB) nasale ou naso-sinusienne est rare et la littérature se limite à des rapports de cas isolés [1,2]. Butt a identifié 35 patients atteints de tuberculose nasale primitive dans une étude approfondie couvrant 95 ans et publié en 1977 [5]. Moon *et al*. ont signalé que parmi 220 cas de tuberculose de la tête et du cou, il n’y a que deux cas qui concerne les cavités naso-sinusiennes [6]. Notre revue de littérature indexée de langue anglaise sur PubMed / MEDLINE a trouvé que 41 cas de localisations naso-sinusiennes ont été rapportés depuis 2000 avec une fréquence élevée pour le sinus maxillaire et l’ethmoïde. La rareté de l’atteinte naso-sinusienne est attribuée aux caractéristiques de la muqueuse nasale: la protection mécanique est assurée par les mouvements ciliaires, les propriétés bactéricides des sécrétions nasales ainsi que la richesse lymphatique de la muqueuse pituitaire qui s’opposent au développement du bacille de koch (BK). Mais, certains facteurs locaux (traumatismes, rhinite atrophique chronique) ou généraux (mauvaises conditions d’hygiène, immunodépression) favoriseraient le développement du BK [2].

Les signes d’appel sont une obstruction nasale, un mouchage mucopurulent, des épistaxis récidivantes associées à une polyadénopathie cervicale. L’atteinte est souvent unilatérale mais des formes bilatérales sont décrites dans plus de 30% des cas [5]. Beltran *et al*. ont proposé que le diagnostic de tuberculose naso-sinusienne soit basé sur les critères suivants: absence de réponse clinique aux antibiotiques empiriques, présence des lésions granulomateuses spécifique dans les prélèvements anatomopathologiques, et l’identification du *Mycobacterium tuberculosis* dans l'échantillon chirurgical [7]. La topographie et l’extension des lésions doivent être évaluées par une échographie, une tomodensitométrie, ou une imagerie par résonance magnétique. L’imagerie par résonance magnétique constitue le meilleur examen pour la visualisation de tuberculose naso-sinusienne car les lésions intéressent plutôt les tissus mous que les tissus squelettiques [8]. L’aspect morphologique à l'endoscopie est plus souvent exophytique qu’ulcératif [9]. L’examen direct ne met généralement pas en évidence de bacilles et la culture est le plus souvent négative [5].

Le diagnostic est histologique par la mise en évidence de granulomes épithélioides et giganto-cellulaires associés à des foyers de nécrose caséeuse. La présence de la nécrose caséeuse est pathognomonique de la tuberculose et élimine de ce fait d’autres diagnostics tels la maladie de Wegener ou la sarcoïdose. Le diagnostic de la tuberculose dans notre observation était retenu par l’étude histologique, et aussi par l’étude bactériologique du matériel intra-sinusien malgré le caractère pauci-bacillaire de la localisation naso-sinusienne. Dans les cas litigieux, on a recours à l’amplification des acides nucléiques (PCR) avec mise en évidence de l’ADN bactérien mais cette technique n'est pas réalisée dans notre contexte [8]. Le traitement est médical. C’est une poly antibiothérapie associant rifampicine, isoniazide, ethambutol et pyrazinamide. Le traitement chirurgical n’a pas sa place dans cette localisation [5]. Non traitée, la tuberculose naso-sinusienne se complique de perforation septale, de rhinite atrophique ou de sténose nasale. L’évolution sous traitement est en général bonne. Cependant, notre patient a présenté une rechute précoce bien qu’elle prenait régulièrement son traitement avec une bonne observance.

Nous avons effectué une revue de littérature en anglais sur la base des données MEDLINE en utilisant «relapse of sinonasal tuberculosis» et «recurrence of sinonasal tuberculosis», et nous n’avons trouvé aucune observation qui signale une récidive de cette localisation naso-sinusienne. La rechute dans notre observation était expliquée par un sous dosage en rifampicine. L’adjonction d’une dose supplémentaire de la rifampicine a permis d’obtenir un dosage sérique de cette molécule dans la fourchette thérapeutique, et a permis de guérir la maladie. La rifampicine à forte dose a été également utilisée par Seijger *et al*. chez un groupe spécifique des patients et ils recommandent son utilisation en cas de sous dosage confirmée en rifampicine, en cas de rechute TB, en cas de suspicion de malabsorption gastro-intestinale ou en cas réponse clinique retardée [10].

## Conclusion

La tuberculose naso-sinusienne est exceptionnelle. Le diagnostic nécessite une vigilance particulière étant donné la rareté de cette localisation et le caractère non spécifique des symptômes cliniques. Nous suggérons d’effectuer un dosage sérique aux antibacillaires devant les rechutes inexpliquées surtout dans les formes rares qui sont généralement paucibacillaire. L’administration de la rifampicine à des fortes doses aux patients présentant un sous dosage en rifampicine permet d’améliorer les résultats du traitement avec un traitement généralement bien toléré.
